# Supplementary material for: GM-CSF engages multiple signaling pathways to enhance pro-inflammatory cytokine responses in human monocytes during Legionella infection
Source: Infect Immun. 2025 Jun 5;93(7):e00565-24. doi: 10.1128/iai.00565-24 (PMC12234439; doi:10.1128/iai.00565-24)
Supplement: Supplemental material — Fig. S1 to S5. [file iai.00565-24-s0001.pdf]

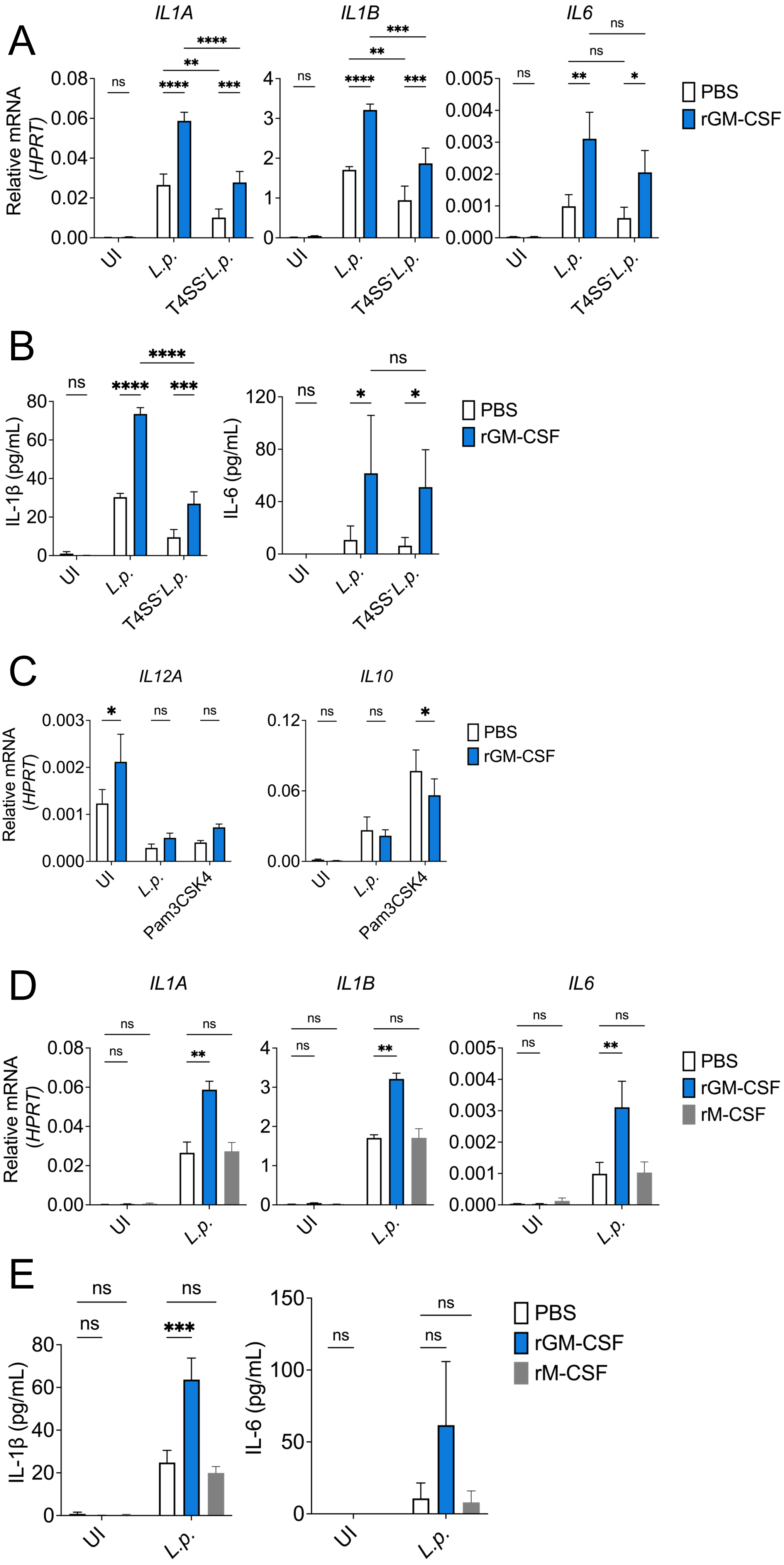
A
IL1A
IL1B
IL6
Relative mRNA (HPRT)
0.08
0.06
0.04
0.02
0.00
4
3
2
1
0
0.005
0.004
0.003
0.002
0.001
0.000
ns
****
**
***
UI
L.p.
T4SS⁻L.p.
PBS
rGM-CSF
B
IL-1β (pg/mL)
IL-6 (pg/mL)
80
60
40
20
0
120
80
40
C
IL12A
IL10
0.003
0.002
0.001
0.000
0.12
0.06
0.00
*
Pam3CSK4
D
rM-CSF
E
150
100
50

Figure S1 (Related to Figure 1): GM-CSF promotes IL1A and IL1B expression in a T4SS-dependent manner without broadly enhancing cytokine expression, a response not induced by M-CSF. THP-1 human monocytes were pre-treated with (A to E) PBS, (A to E) rGM-CSF, or (D and E) M-CSF for 30-60min. Cells were then left (A to E) uninfected (UI), infected with (A to E) *L.p.* or (A and B) $T4SS^{-}$ *L.p.* or treated with the (C) TLR2 agonist Pam3CSK4. (A, C, and D) Cells were harvested at 6hr post-infection (hpi) to measure (A and D) *IL1A*, *IL1B*, and *IL6* or (C) *IL12A* and *IL10* transcript levels by qPCR, or (B and E) cells and supernatants harvested at 24hr after infection to measure IL-1β and IL-6 release by ELISA. Data represent the mean $\pm$ SEM of triplicate wells from at least three independent experiments. Data were analyzed by two-way ANOVA with Sidak's multiple comparisons test; ****, $P < 0.0001$; ***, $P < 0.001$; **, $P < 0.01$; *, $P < 0.05$; ns, not significant.

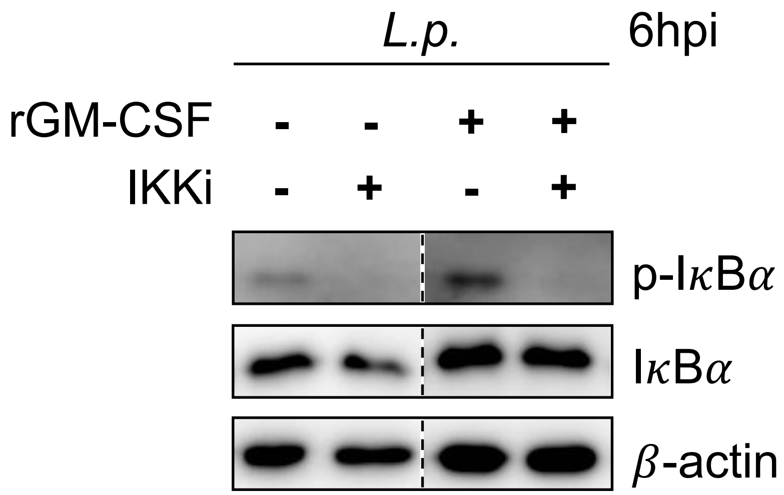

Figure S2 (related to Figure 2): IKK inhibitor abrogates *Legionella*-induced IκBα phosphorylation in THP-1 human monocytes. THP-1 monocytes were pre-treated with vehicle control or the IKK inhibitor BMS-345541 for 1hr. Cells were then treated with PBS or rGM-CSF for 1hr and then left uninfected (UI) or infected with *L.p*. Cells were harvested at 6hr post-infection (hpi) to perform immunoblot analysis for phospho-IκBα, total IκBα, or β-actin as loading control. Lanes from one membrane have been cropped and moved to depict the appropriate conditions. No changes were made to the original image during the editing.

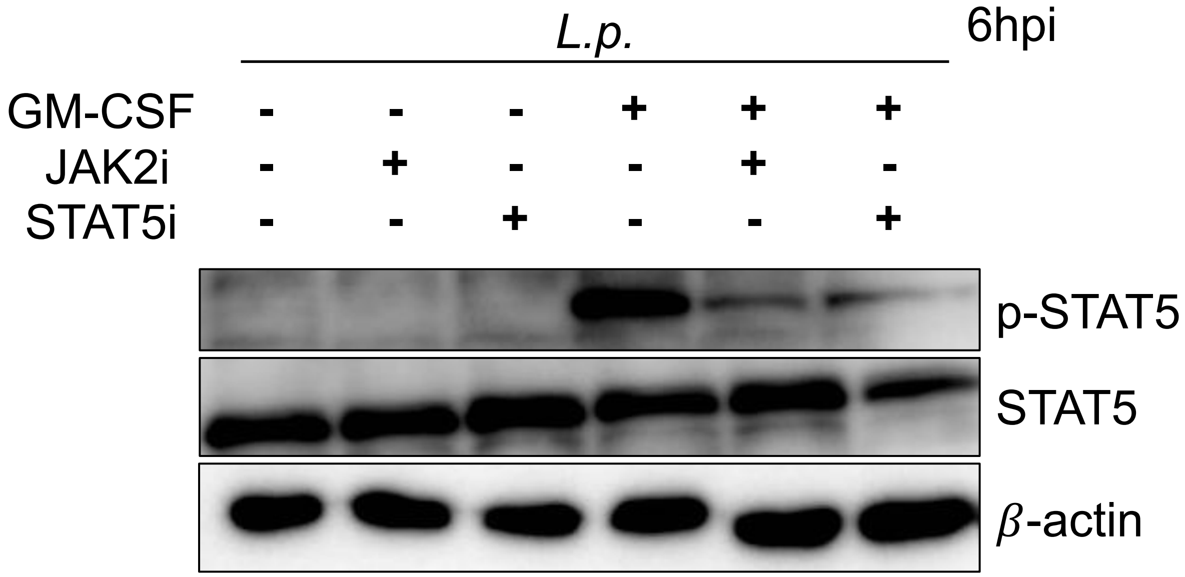

Figure S3 (Related to Figure 3): STAT5 and JAK2 inhibitors abrogate GM-CSF-dependent STAT5 phosphorylation in THP-1 human monocytes infected with *Legionella*. THP-1 monocytes were pre-treated with vehicle control, the JAK2 inhibitor NVP-BSK805, or the STAT5 inhibitor SH 4-54 for 1hr. Cells were then treated with PBS or rGM-CSF for 30-60min and then left uninfected (UI) or infected with *L.p*. Cells were harvested at 6hr post-infection (hpi) to perform immunoblot analysis for phospho-STAT5, total STAT5, or β-actin as loading control.

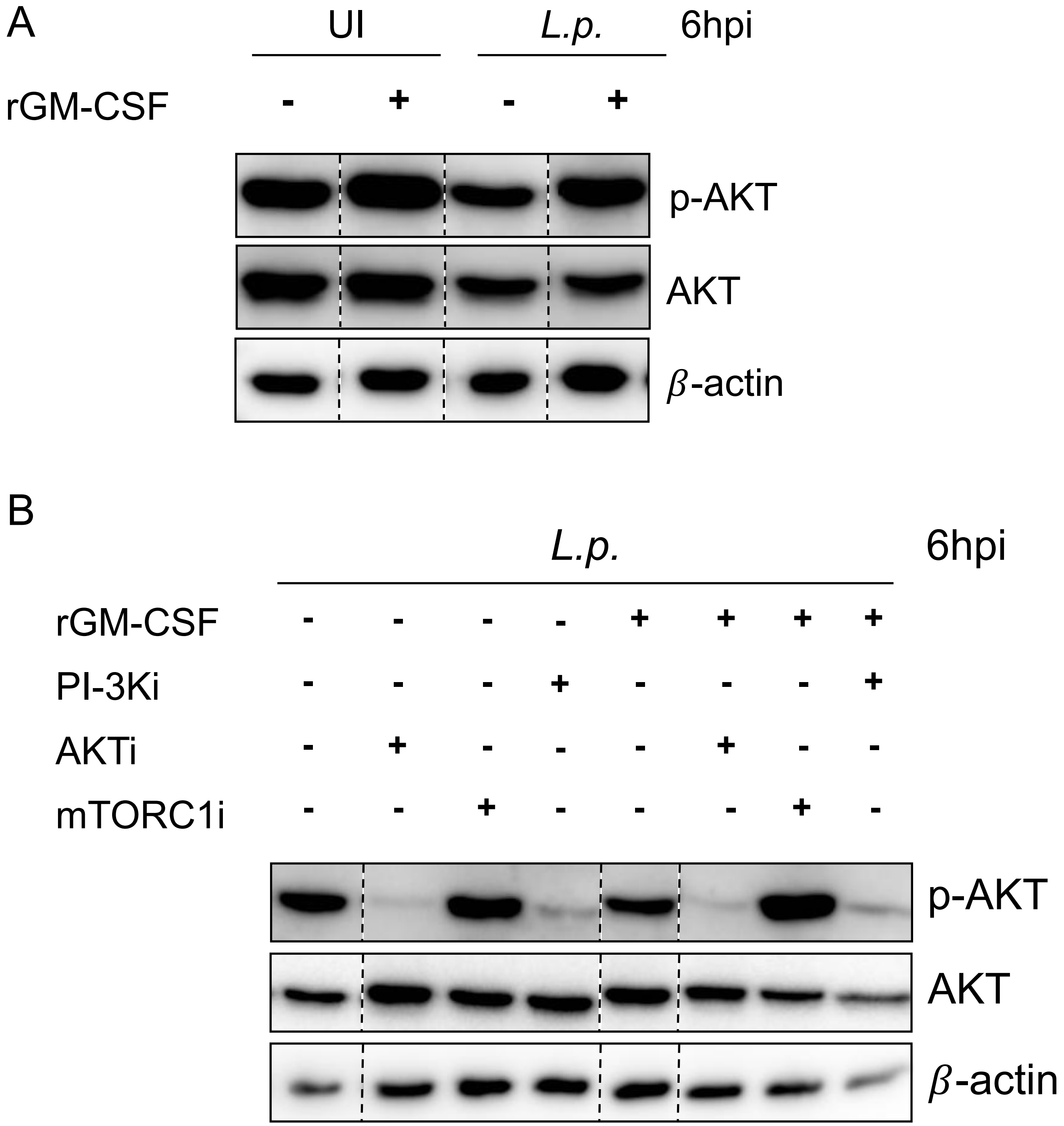
A
UI
L.p.
6hpi
rGM-CSF - + - +
p-AKT
AKT
β-actin
B
L.p.
6hpi
rGM-CSF - - - - + + + +
PI-3Ki - - - + - - - +
AKTi - + - - - + - -
mTORC1i - - + - - - + -
p-AKT
AKT
β-actin

Figure S4 (Related to Figure 4): THP-1 human monocytes exhibit basal Akt phosphorylation that is downregulated by PI-3K and AKT inhibitors. (A) THP-1 human monocytes were pre-treated with PBS or rGM-CSF for 1hr. Cells were harvested at 6hr after infection to perform immunoblot analysis for phospho-Akt, total Akt, or β-actin as loading control. Lanes from one membrane have been cropped and moved to depict the appropriate conditions. No changes were made to the original image during the editing. (B) THP-1 monocytes were pre-treated with vehicle control, PI3-K inhibitor Ly294002, Akt inhibitor MK-2206 or mTORC1 inhibitor Rapamycin for 1hr. Cells were then treated with PBS or rGM-CSF for 1hr and then left uninfected (UI) or infected with *L.p*. Cells were harvested at 6hr post-infection (hpi) to perform immunoblot analysis for phospho-Akt, total Akt, or β-actin as loading control. Lanes from one membrane have been cropped and moved to depict the appropriate conditions. No changes were made to the original image during the editing.

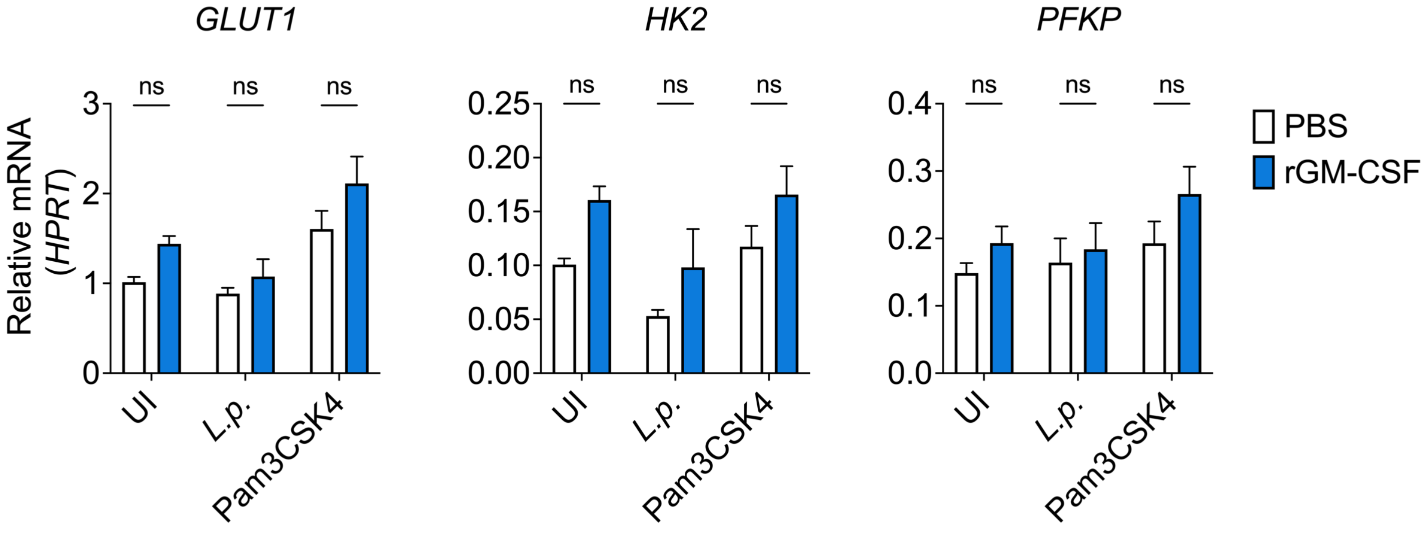

Figure S5 (Related to Figure 5): GM-CSF does not significantly upregulate rate-limiting glycolytic genes in *Legionella*-infected human monocytes. THP-1 monocytes were pre-treated with PBS or rGM-CSF for 30-60min. Cells were then left uninfected, infected with *L.p.* or treated with the TLR2 agonist Pam3CSK4. Cells were harvested at 6hr after infection to measure *GLUT1, PFKP,* and *HK2* transcript levels by qPCR. Data represent the mean ± SEM of triplicate wells from at least three independent experiments. Data were analyzed by two-way ANOVA with Sidak's multiple comparisons test; ns, not significant.
